# Sociodemographic Determinants of Knowledge, Attitudes, and Practices Toward Hepatitis B Infection Among Pregnant Women: A Cross-National Study in Jordan

**DOI:** 10.7759/cureus.58053

**Published:** 2024-04-11

**Authors:** Nader Alaridah, Sara Abu-Mutaw', Ghayda Abu-Assaf, Leen Al Dwikat, Raba’a F. Jarrar, Haneen O Abuhani, Basmalah Al-Hawadi, Saif Alhawadi, Mohammad Al Shdifat, Rayan M Joudeh

**Affiliations:** 1 Department of Pathology, Microbiology, and Forensic Medicine, The University of Jordan, Amman, JOR; 2 College of Medicine, The University of Jordan, Amman, JOR; 3 Department of Clinical Laboratory Sciences, School of Science, The University of Jordan, Amman, JOR; 4 College of Medicine, Faculty of Medicine, Al-Balqa Applied University, Amman, JOR; 5 College of Medicine, Sulaiman Alrajhi University, Al-Bukayriyah, SAU

**Keywords:** jordan, pregnant women, practice, attitude, knowledge, hbv

## Abstract

Background

Mother-to-child transmission (MTCT) of the hepatitis B virus (HBV) is significant, as most infants infected at birth go on to develop chronic hepatitis B. Vaccination and antiviral treatment during pregnancy could primarily prevent vertical transmission. Therefore, the purpose of this study is to assess pregnant Jordanian females’ knowledge, attitude, and practices (KAP) toward HBV. In addition, to explore the relationship between the level of KAP toward HBV infection and its predictors.

Methods

Our cross-sectional study was conducted among pregnant women in Jordan. We enrolled 621 participants between January and April 2023. Our survey was derived from a previously validated tool that was used to investigate a similar aim as our study. The survey was done via Google Forms (Google LLC, Mountain View, California, United States) and it contained questions divided into four main sections: participants’ demographics, knowledge section, attitudes section, and practices section.

Results

The majority of participants have neither a personal nor family history of HBV, and only 91 (14.7%) of the participants had a medical degree. The overall knowledge, attitude, and practice (KAP) scores were low, as only 176 (28.3%), 315 (50.7%), and 244 (39.3%) of participants achieved high levels of knowledge, attitude, and practice scores, respectively. A significant association was found between knowledge level, practice assessment, and the following variables: age, educational level, job, study field, history of HBV in the family, and source of knowledge. Regarding attitude, a significant association was found with the job, study field, and source of knowledge.

Conclusion

This study found that pregnant women in Jordan had a low level of awareness of HBV infection. Thus, more efforts should be made to raise awareness about HBV among high-risk groups, especially pregnant women.

## Introduction

Globally, the hepatitis B virus (HBV) is a serious health problem, with 1.5 million new cases of chronic hepatitis B infection occurring annually, impacting 296 million people worldwide [1]. The global epidemiology of HBV is demonstrated in Middle Eastern countries, with intermediate (2-5%) to high endemicity (>5%) chronic carriers in many countries [1]. The majority of the cases are transmitted from mother to child during pregnancy or after birth [2]. Mother-to-child transmission (MTCT) of HBV is significant, as most infants infected at birth go on to develop chronic hepatitis B [3]. The chronicity depends mainly on age, as the risk of infants becoming chronic carriers if infected at birth is 70-90%, whereas the chance of becoming a chronic carrier is only 5% in adult-infected individuals [3]. On the other hand, adult infection usually causes an acute, self-limiting illness followed by viral clearance or, in rare cases, fulminant liver failure [4]. In contrast, infants’ infection with the virus results in a life-long chronic infection and virus carriage [5]. The hepatitis B surface antigen (HBsAg) can be carried without causing any symptoms, or the condition can progress to severe, active variants that cause fibrosis, liver cirrhosis, and hepatocellular carcinoma (HCC) [4]. In Jordan, the prevalence of HBV among pregnant women is around 5% [6], which is less than in China and some African countries (6-11.7%) [7-9], but quite higher than in other Middle Eastern countries (1.5-4%) [10,11]. As vertical transmission is the main route for HBV infection among pregnant women, it could be prevented mainly by vaccination and antiviral treatment during pregnancy [12]. The first step in prevention is to screen all pregnant women in their first trimester to identify the best management step for the mother and to prevent vertical transmission to her baby [13]. The HBV vaccine is safe and effective even during pregnancy, and all infected or non-immune (anti-HB levels lower than 10 mIU/mL) pregnant women should be vaccinated against HBV, whether they are at high risk for the virus or not [14]. However, 10% of infants of HBV-infected mothers still develop HBV infection despite vaccination and administration of hepatitis B immune globulin (HBIG) [13]. Additional measures should be taken for the prevention of MTCT, such as the use of antiviral therapy (lamivudine, telbivudine, or tenofovir) with HBeAg-positive mothers, in addition to the standard infant immune prophylaxis (the first dose of HBV vaccine and HBIG) at birth [15]. This will effectively reduce the vertical transmission of HBV and avoid many of its significant future consequences [15]. In light of the WHO's effort to achieve the elimination of hepatitis infection by 2030, promoting awareness among the general population as well as high-risk groups such as pregnant women is crucial. Numerous studies have been conducted among pregnant women [6,10,16-18]. They found the awareness among pregnant women to be unsatisfactory. Thus, there is a need to take immediate action to control the increase in the prevalence of HBV among pregnant women, provide welfare for the infant, and prevent sequelae of HBV infection. Therefore, this study aims to investigate pregnant Jordanian females’ knowledge, attitudes, and practices toward HBV. In addition, to explore the relationship between the level of knowledge, attitudes, and practices toward HBV infection and its predictors.

## Materials and methods

Our cross-sectional study was conducted among pregnant women in Jordan. We enrolled 621 participants between January and April 2023. The target population was reached through Jordan University Hospital, Al-Zarqa Governmental Hospital, Prince Hamza Hospital, Al Bashir Hospital, Princess Rahma Hospital, As-Salt Hospital, and Nadim Hospital. The selection process for the participants was a stratified, cluster sampling technique, ensuring representation from the population that is visiting the health facility in Jordan. Jordan is usually divided into three regions: North, Central, and South, with a high population density in the Central. We selected the main health facility in each region to recruit an equal number of representatives from each health facility to include an equal population from all sociodemographic levels in every region. After getting ethical permission, we contacted administrators from each institution to help disseminate the questionnaire to eligible individuals. Pregnant women who were attending the gynecology/obstetric clinic were invited to participate in the study. The questionnaire was distributed to all participants via text message, and those who had difficulty accessing the Internet or could not read were invited to in-person interviews. The minimum sample size needed for the study is 385, which was calculated based on 5% marginal error and 50% prevalence. 

Questionnaire administration

Eligible participants were invited to participate voluntarily by filling out the questionnaire. The questionnaire was sent to the participants through an online survey link. Informed consent was obtained from all participants. It was explicitly mentioned that participants could withdraw their responses freely at any time. Participants were requested to submit the necessary information to obtain reliable results, but no personal information was gathered.

Measurement tool

Our survey was derived from a previously validated tool that was used to investigate a similar aim as our study [19]. The survey was done via Google Forms (Google LLC, Mountain View, California, United States), and it contained questions divided into four main sections. The first section assessed participants’ demographics, followed by a knowledge section with 20 items, an attitudes section with seven items, and a practices section with eight items. A pilot test was done on the questionnaire to make sure the questions were clear and comprehensible before being distributed and used. Participants should achieve a score of more than 75% to be considered to have an appropriate level of knowledge, attitudes, or practices. The questionnaire is shown in Appendix 1, 2, and 3.

Ethical considerations

The protocol was established in accordance with the Helsinki Declaration's ethical principles, and it had been reviewed and accepted by the Institutional Review Board (IRB) at the University of Jordan under reference number 0/2022/2506 in meeting no. 2022/24. Before completing the questionnaire, all individuals gave written informed consent. The data was collected and processed in an anonymous form before being stored on a personal computer that only the authors have access to.

Statistical analysis

Microsoft Excel 2016 (Microsoft Corporation, Redmond, Washington, United States) was used to enter the data, which was then imported into IBM SPSS Statistics for Windows, Version 25 (Released 2017; IBM Corp., Armonk, New York, United States) for analysis. For each numerical and categorical variable, descriptive statistics were calculated and reported as frequency and percentage, or mean and standard deviation, respectively. The Chi-square test was used to assess the relationship between demographic factors, knowledge, attitudes, and practices. Multivariate regression analysis was used to evaluate each independent variable after controlling for possible confounders. Each correct response received one point. If a participant correctly responded to 75% of the questions or more in each KAP section, the score was deemed to be good. If less than 75% of the questions in each section were correctly answered, the participant's score was deemed poor.

## Results

Demographics of survey participants

The demographics of the participants are presented in Table 1. Of the total 621 women, N = 299 (48.1%) were between 28-37 years of age, and nearly N = 477 (76.8%) of the participants live in the city; N = 564 (90.8%) of them were of high education. Of the participants, N = 91 (14.7%) had a medical study field. There were nearly equal numbers of employed and unemployed participants. Almost all participants, N = 616 (99.2%), don’t have a history of HBV, and N = 537 (86.5%) of them don’t have a history of HBV in their family. Their source of knowledge varies between medical N = 126 (20.3%), non-medical N = 311 (50.1%), and never heard of it N = 184 (29.6%).

**Table 1:** Demographics of survey participants HBV: hepatitis B virus

	Answer	Count	%
Age	18-27	235	37.8%
	28-37	299	48.1%
	More than 38	87	14%
Education	High education	564	90.8%
	Low education	57	9.2%
Job	No work	306	49.3%
	Work	315	50.7%
Study field	Medical	91	14.7%
	Non-medical	252	40.6%
	None	278	44.8%
Residence	Urban	477	76.8%
	Rural	144	23.2%
History of HBV in family	Yes	84	13.5%
	No	537	86.5%
History of HBV	Yes	5	0.8%
	No	616	99.2%
Source of knowledge	Medical	126	20.3%
	Non medical	311	50.1%
	Never heard	184	29.6%

Knowledge of the hepatitis B virus

Concerning knowledge assessment towards HBV, as shown in Table 2, N = 516 (83.1%) of participants have heard of hepatitis, and N = 424 (68.3%) have heard of hepatitis B specifically. Out of all participants, N = 374 (60.2%) thought hepatitis B was a viral disease; N = 497 (80%) believed it affected liver function; N = 258 (41.5%) assumed it caused liver disease; and N = 364 (58.5%) considered it could affect any age group. Regarding its symptoms, only N = 208 (33.5%) considered them like early flu and fever; N = 436 (70.2%) thought jaundice was common; N = 329 (53%) answered nausea and vomiting as common symptoms; and N = 228 (36.7%) believed it could be asymptomatic. Thoughts on routes of transmission of the virus varied between unsterilized needles (N = 409, 65.9%), contaminated blood (N = 423, 68.1%), nose-piercing (N = 327, 52.7%), unsafe intercourse (N = 280, 45.1%), mother-to-child (N = 302, 48.6%), and food prepared by infected individuals (N = 149, 24%). Only N = 74 (11.9%) recognized the disease as untreatable; N = 144 (23.2%) thought it could be self-cured by the body. However, N = 399 (64.3%) of the respondents believed there was an available vaccine.

**Table 2:** Knowledge assessment

	Answer	Count	%
Have you ever heard of a disease termed hepatitis?	Yes	516	83.1%
Have you ever heard of a disease termed hepatitis B?	Yes	424	68.3%
Is hepatitis B a viral disease?	Yes	374	60.2%
Can hepatitis B affect liver function?	Yes	497	80%
Can hepatitis B cause liver cancer?	Yes	258	41.5%
Can hepatitis B affect any age group?	Yes	364	58.6%
The early symptoms of hepatitis B are the same as cold and flu fever running nose?	Yes	208	33.5%
Jaundice is one of the common symptoms of hepatitis B	Yes	436	70.2%
Are nausea, vomiting, and loss of appetite common symptoms of hepatitis?	Yes	329	53%
Are there no symptoms of hepatitis B in some of the patients?	Yes	228	36.7%
Can hepatitis B be transmitted by unsterilized syringes needles and surgical equipment?	Yes	409	65.9%
Can hepatitis B be transmitted by contaminated blood and blood products?	Yes	423	68.1%
Can hepatitis B be transmitted by using blades of the barber ear and nose piercing?	Yes	327	52.7%
Can hepatitis B be transmitted by unsafe sex?	Yes	280	45.1%
Can hepatitis B be transmitted from mother to child?	Yes	302	48.6%
Can hepatitis B be transmitted by contaminated water/food prepared by a person suffering from these infections?	No	149	24%
Is hepatitis B curable/treatable?	No	74	11.9%
Can hepatitis B be self-cured by the body?	Yes	144	23.2%
Is vaccination available for hepatitis B?	Yes	399	64.3%
Is a specific diet required for the treatment of hepatitis B?	No	98	15.8%

Attitudes toward the hepatitis B virus

Table 3 shows the attitudes among participants. Most of the participants, N = 374 (60.2%), denied they could get the disease, and almost half, N = 300 (48.3%), would experience fear if that happened. Almost two-thirds of respondents (N = 473, 76.2%) chose to talk to a physician about their illness, and 516 (83%) would refer to him if they had symptoms. Around N = 561 (90.3%) were willing to go to a health facility as soon as they realized such symptoms. When asked how expensive the treatment is, N = 248 (39.9%) didn’t know, N = 75 (12.1%) thought it would be free, and only N = 98 (15.8%) believed it was expensive. Around N = 338 (54.4%) of the participants feared it would spread to their families.

**Table 3:** Attitude assessment

Question	Answer	Count	%
Do you think you can get hepatitis B?	No	374	60.2%
	Yes	247	39.8%
What would be your reaction if you found that you have hepatitis B?	Fear	300	48.3%
	Shame	11	1.8%
	Surprise	182	29.3%
	Sadness	128	20.6
Who would you talk to about your illness?	Physician	473	76.2%
	Spouse	104	16.7%
	Parents	35	5.6%
	Child	2	0.3%
	Other relatives	7	1.1%
What will you do if you think that you have symptoms of hepatitis B?	Go to a health facility	98	15.8%
	Go to a doctor	516	83.1%
	Go to a traditional healer	4	0.6%
	Will not go to a physician	3	0.5%
If you had symptoms of hepatitis B, at what stage would you go to the health facility?	Own treatment fails	22	3.5%
	After 3-4 weeks of the appearance of symptoms	35	5.6%
	As soon as I realized the symptoms are of hepatitis B	561	90.3%
	Will not go to a physician	3	0.5%
How expensive do you think the diagnosis and treatment of hepatitis B?	Free	75	12.1%
	Reasonable	86	13.8%
	Somewhat expensive	114	18.4%
	Expensive	98	15.8%
	Don't know	248	39.9%
What worries you most if you will be diagnosed with hepatitis B?	Fear of death	207	33.3%
	Fear of disease spread to family	338	54.4%
	Cost of treatment	41	6.6%
	Isolation from the society	35	5.6%

Practice toward hepatitis B virus

**Figure 1:**
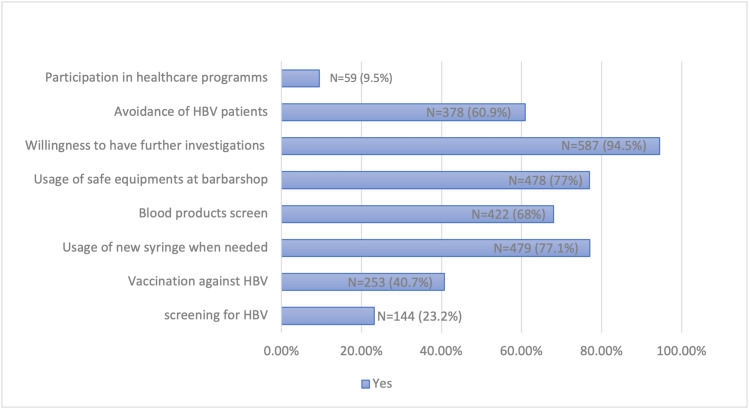
Practice assessment

Certain practices were assessed, and the results are shown in Figure 1. Only N = 144 (23.2%) have done screening for hepatitis B, and N = 253 (40.7%) got vaccinated. Regarding safety measures, N = 479 (77.1%) asked for a new syringe before use, N = 422 (68%) asked for screening of blood before transfusion, and N = 478 (77%) asked a barber to change equipment. Almost all participants, N = 587 (94.5%), would undergo further investigations in the event of the diagnosis of the disease. Around N = 378 (60.9%) responded to avoiding meeting hepatitis B patients. Only N = 59 (9.5%) participated in health education programs related to hepatitis.

Associated factors toward knowledge, attitude, and practices toward HBV infection

As shown in Table 4, a significant association was found between knowledge level, practice assessment, and the following variables: age, educational level, job, study field, history of HBV in the family, and source of knowledge. Regarding attitude, a significant association was found with the job, study field, and source of knowledge.

**Table 4:** Association between demographic characteristics and knowledge, attitudes, and practices toward HBV * Denotes significance; HBV: hepatitis B virus

Covariates		Knowledge			Attitude			Practice		
		Low	High		Low	High		Low	High	
		Count	Count	P-value	Count	Count	P-value	Count	Count	P-value
Age	18-27	183	52	0.001*	128	107	0.127	139	96	0.001*
	28-37	193	106		137	162		169	130	
	More than 38	69	18		41	46		69	18	
Educational level	Higher	394	170	0.002*	272	292	0.1	326	238	0.000*
	Lower	51	6		34	23		51	6	
Job	No work	254	52	0.000*	170	136	0.002*	210	96	0.000*
	Work	191	124		136	179		167	148	
Study field	Medical	21	70	0.000*	21	70	0.000*	23	68	0.000*
	Non-medical	191	61		128	124		167	85	
	None	233	45		157	121		187	91	
Residence	Urban	315	162	0.000*	235	242	0.993	296	181	0.21
	Rural	130	14		71	73		81	63	
History of HBV in family	Yes	35	49	0.000*	45	39	0.397	30	55	0.000*
	No	410	127		261	276		347	190	
History of HBV	Yes	4	1	0.678	2	3	0.677	2	3	0.341
	No	441	175		304	312		375	241	
Source of knowledge	Medical	35	91	0.000*	45	81	0.001*	32	94	0.000*
	Non-medical	410	85		261	234		345	150	

Logistic regression analysis between demographic characteristics and knowledge, attitudes, and practices toward HBV

As shown in Table 5, there is a statistically significant association between the level of knowledge and the following variables: job: work (odds ratio (OR) = 2.67; p<0.04; confidence interval (CI) = 1.03-6.9), reference: no work; study field: medical (OR = 3.76; p<0.000; CI = 1.82-7.74), reference: non-medical; residence: city (OR = 3.2; P<0.02; CI = 1.52-6), reference: village; history of HBV in the family: yes (OR = 7.55; p<0.000; CI = 4.13-13.2), reference: no; source of knowledge: medical (OR = 7.53; p<0.000; CI = 4.13-13.72). Regarding participants’ attitudes, a statistically significant association was found with the study field (OR = 3.7, P<0.000, CI = 0.47-2.02), reference: non-medical. In regards to practice assessment, there is a statistically significant association with the following factors: age: 18-27 (OR = 3.23; P<0.001; CI = 1.65-6.35), 28-37 (OR = 2.42; P<0.007; CI = 1.27-4.59), reference: 38+; educational level: high (OR = 4.54; P<0.001; CI = 1.8-11.34), reference: low; job: work (OR = 4.73; P<0.001; CI = 1.8-11.88), reference: no work; study field: medical (OR = 2.21; P<0.027; CI = 1.09-4.45), none: (OR = 4.3; P< 0.003; CI = 1.66-11.14), reference: non-medical; history of HBV in family: yes (OR = 4.42; P<0.000; CI = 2.61-7.49), reference: no; source of knowledge: medical (OR = 4.9; P<0.000; CI = 2.71-8.8), reference: non-medical.

**Table 5:** Logistic regression analysis between demographic characteristics of knowledge, attitudes, and practices toward HBV OR: odds ratio; CI: confidence interval; P: P-value; NA: not applicable; not included in the logistic regression analysis; * denotes significance

Covariates		Knowledge			Attitude			Practice		
		OR	CI	p-value	OR	CI	p-value	OR	CI	p-value
Age	18-27	1.26	0.59-2.7	0.55	NA			3.23	1.65-6.35	0.001*
	28-37	1.4	0.69-2.83	0.35				2.42	1.27-4.59	0.007*
	More than 38	Reference						Reference		
Education level	High	1.67	0.6-4.55	0.32	NA			4.54	1.8-11.34	0.001*
	Low	Reference						Reference		
Job	Work	2.67	1.03-6.9	0.04*	1.26	0.62-2.56	0.53	4.73	1.88-11.88	0.001*
	No work	Reference			Reference			Reference		
Study field	Medical	3.76	1.82-7.74	0.000*	3.7	1.87-7.31	0.000*	2.21	1.09-4.45	0.027*
	None	1.97	0.72-5.37	0.18	0.98	0.47-2.015	0.95	4.3	1.66-11.14	0.003*
	Non-medical	Reference			Reference			Reference		
Residence	Urban	3.02	1.52-6	0.002*	NA			NA		
	Rural	Reference								
History of HBV in family	Yes	7.55	4.31-13.2	0.000*	NA			4.42	2.61-7.49	0.000*
	No	Reference						Reference		
Source of knowledge	Medical	7.53	4.13-13.72	0.000*	0.92	0.53-1.58	0.75	4.9	2.71-8.85	0.000*
	Non-medical	Reference			Reference			Reference		

## Discussion

This cross-national, multicenter study in Jordan aimed to assess knowledge, attitudes, and practices toward HBV among pregnant women and its predictors. Overall, two-thirds of pregnant women had a low level of knowledge about HBV, especially among those who don’t work, have no medical study background, live in a rural area, and have no family history of HBV. This unsatisfactory level of knowledge is consistent with other studies [6,10,17,18,20,21]. The majority of respondents were aware of the terms hepatitis and hepatitis B and acknowledged it’s a viral disease, which was similar to a study conducted in Saudi Arabia [10], in contrast to another study conducted in Jordan [6]. This result could be due to the high socioeconomic status of our participants. Also, we noticed the major gaps in certain areas of knowledge; as reported, in the era of disease presentation, only one-third were aware that it can present with flu-like symptoms or no symptoms at all. In addition, less than half of respondents believe that HBV can cause liver cancer as a long-term complication. Obtaining sufficient disease-related knowledge may allow people to make proper decisions by visiting a doctor immediately when symptoms start and before a complication occurs. Moreover, improving illness awareness would minimize patient worry, increase treatment compliance and satisfaction, and lower treatment costs [22-24].

In terms of awareness about the mode of transmission, participants showed average results, in which more than half of them were aware that HBV can be transmitted by unsterilized syringes, contaminated blood products, and the reuse of blades and nose-piercing needles. On the other hand, less than half of them were aware that it can be transmitted vertically from mother to child and through unsafe sex. Surprisingly, only one-fourth of women asserted that HBV can’t be transmitted by food or water prepared by a person infected with HBV. This major defect in knowledge of transmission routes was also observed in other studies [10,17,18,20,21]. As reported by a study conducted in Turkey, increased knowledge of the disease's transmission is associated with a lower risk of getting hepatitis B. Furthermore, high levels of awareness about the prevention and spread of hepatitis B are important for encouraging testing and identifying people who are affected. [25] In addition, the majority of the participants claimed that there is a cure for HBV or that it can be cured by itself; this false belief was also reported in a study conducted in China [20]. Moreover, 40% of participants underestimated treatment costs. The level of attitudes among the participants is considered moderate, as N = 315 (50.7%) of women had a good attitude towards HBV. This finding is similar to a study conducted in Saudi Arabia [10], however, the attitude was reported to be low in countries like Ethiopia and Vietnam [17,21].

Our study disclosed that most participants would go first to a doctor as soon as symptoms of HBV infection appear, and around three-quarters would like to talk about their illness with their doctors, while the remaining would like to talk with a family member (spouse, parents, or other relatives). In addition, more than half of the participants would feel some sort of stigma if they were diagnosed with HBV, as they would feel shame, surprise, or sadness. Similarly, a study in Vietnam revealed high levels of stigma among pregnant women [17]. This level of stigma was reflected in the attitudes of the respondents, as they were worried about being in contact with HBV-infected patients; in fact, around 90% of the participants would go to a healthcare facility as soon as they realized the symptoms of hepatitis B, and a small proportion of them had the fear of being isolated if the diagnosis was confirmed. It is commonly acknowledged that HBV-associated stigma can significantly impact health-seeking behaviors, including screening, prevention, diagnosis, and treatment of HBV [26]. Regarding practice assessment, the majority of the participants expressed poor practice measures towards HBV infection. Surprisingly, less than half got vaccinated against HBV, and only one-quarter had done screening for it. Vaccination plays an important role in providing protection and decreasing complications of HBV, as can be seen in Libya and Jordan in a practical sense, where implementing the vaccine decreased the prevalence of the disease from 2.8 to 1.5% [27] and from 9.9% to 2.4%, respectively [28]. In our study, only 40% of participants had gotten the vaccine, which was similar to other population groups in Jordan [16]. Regarding HBV prevention, participants showed better practices, as they would ask for new equipment during medical procedures, during their visit to the barbershop, or while getting an ear/nose piercing. Around ten percent of the pregnant women had attended an educational program about HBV infection; hence, we need more participation in such programs directed at childbearing age women, as they would be future mothers.

As we noticed, having a high degree of education and employment, as well as getting their information from medical sources, were all associated with higher levels of knowledge and practice. Furthermore, higher education levels may help them land a better job. Thus, the respondent will be able to get information from medical sources by scheduling routine checkups with their doctor. This suggests that there is a complicated link between a person's work situation, education level, and capacity to get reliable medical information about hepatitis B. Unlike what was reported by a Chinese study [20], participants with a family history of HBV infection were noted to have higher levels of knowledge and practice. On the other hand, living in a rural area is a predictor of lower knowledge levels, as found in Ethiopia [21]. Also, only those who have a medical background tend to have a high level of attitude. Future public health measures are required to prevent mother-to-child transmission by implementing hepatitis B screening programs. Moreover, it is important to increase the knowledge of HBV among all females of reproductive age, regardless of their socio-economic status, to increase the rate of hepatitis B birth dosage immunization. Raising awareness through programs and interventions could be done accordingly by government officials and pharmaceutical companies. In addition to that, screening programs, free of charge or for a minute fee, should be put in place for all those at risk.

This research faces a few drawbacks. As the survey was designed to recruit participants from pregnant women in the general population, whether they are hepatitis B patients or not, it assessed knowledge, attitudes, and practices toward hepatitis B in pregnant women from the general population, with only a small percentage of them having a history of hepatitis B. Secondly, the survey may have a selection bias; this comes from the method of its distribution using mainly online methods, which limited our selection to a population with certain demographics that have access to the internet; furthermore, recall bias was inventible in self-reported questions. Despite the few drawbacks, the importance of our research lies in its targeted population, as it explored the knowledge, attitudes, and practices towards hepatitis B in pregnant females, who are the most important population of hepatitis B patients, as the prevention of its transmission to newborns and developing chronicity with its disastrous outcomes starts with them.

## Conclusions

In conclusion, more efforts should be made to raise awareness about HBV among high-risk groups, especially among pregnant women, as less than a third of the participants were considered to have a high knowledge level. However, appropriate attitudes and adherence to protective measures were noted among participants. Pregnant women should be informed about the high risk of HBV infection on maternal and fetal health, and more workshops should be done to raise awareness regarding diagnosis and treatment and to implement the importance of vaccination against such infections.

## Figures and Tables

**Figure 2:**
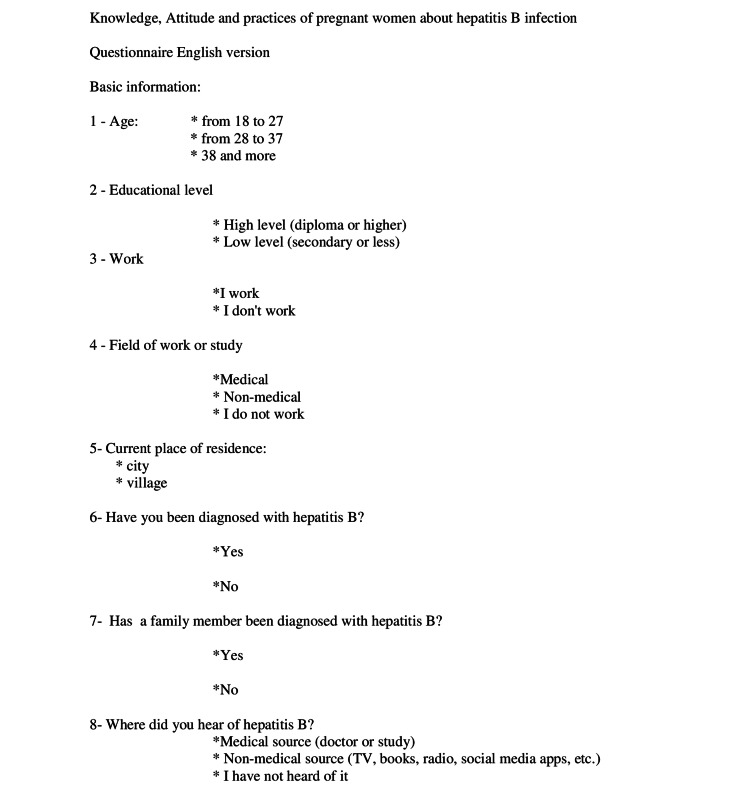
Demographic questions

**Figure 3:**
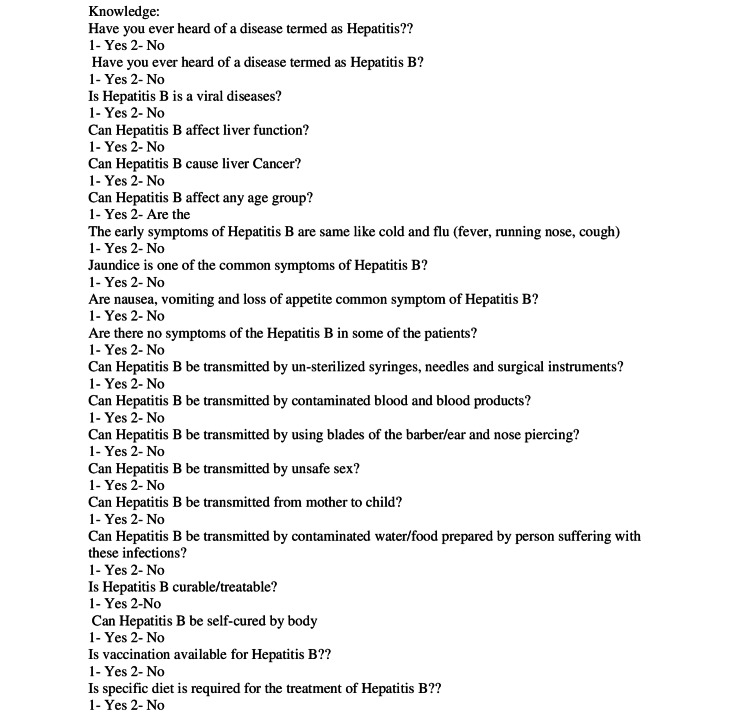
Knowledge questions

**Figure 4:**
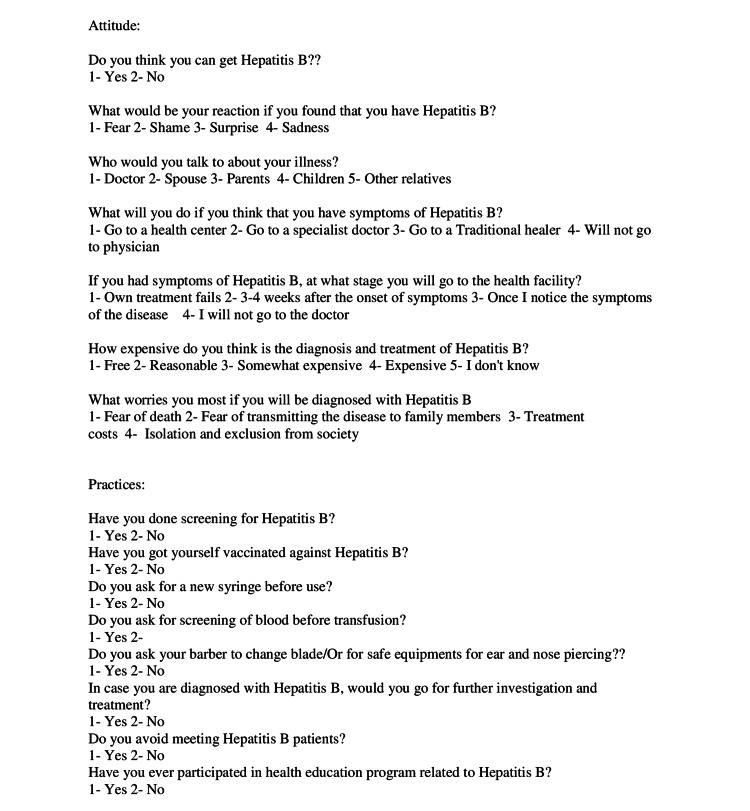
Attitude and practice questions

## Appendices

Appendix 1 

Appendix 2

Appendix 3
